# The role of adipose-derived stem cells-derived extracellular vesicles in the treatment of diabetic foot ulcer: Trends and prospects

**DOI:** 10.3389/fendo.2022.902130

**Published:** 2022-07-27

**Authors:** Hongyan Deng, Yong Chen

**Affiliations:** ^1^ Division of Endocrinology, Internal Medicine, Tongji Hospital, Huazhong University of Science & Technology, Wuhan, China; ^2^ Laboratory of Endocrinology, Tongji Hospital, Huazhong University of Science & Technology, Wuhan, China; ^3^ Branch of National Clinical Research Center for Metabolic Diseases, Hubei, China

**Keywords:** diabetic foot ulcer, extracellular vesicles, adipose tissue-derived stem cells, wound healing, exosomes

## Abstract

Diabetic foot ulcer(DFU) is one of the most severe chronic complications of type 2 diabetes mellitus, which is mainly caused by peripheral vascular occlusion with various degrees of infection. Treatment of DFU is difficult, and ulcer formation in lower limbs and deep-tissue necrosis might lead to disability or even death. Insulin resistance is the major mechanism of type 2 diabetes mellitus development, largely caused by adipose tissue dysfunction. However, adipose tissue was recently identified as an important endocrine organ that secretes bio-active factors, such as adipokines and extracellular vesicles(EVs). And adipose tissue-derived stem cells(ADSCs) are abundant in adipose tissue and have become a hot topic in the tissue engineering field. In particular, EVs derived from ADSCs contain abundant biomarkers and mediators. These EVs exert significant effects on distant cells and organs, contributing to metabolic homeostasis. In this review, we aim to elaborate on the mechanisms of diabetic non-healing wound development and the role of ADSCs-EVs in wound repair, which might provide a new therapy for treating DFU.

## Introduction

Recently, the incidence of diabetes mellitus (DM) worldwide has been steadily increasing because of the growing prevalence of sedentary lifestyles and energy-dense, western dietary change (1). According to the latest report, in 2021, about 537 million adults (aged 20–79 years) worldwide suffered from DM, which means that one in ten people have diabetes, and the number is projected to rise to 11.3% (643 million) by 2030 and to 12.2% (783 million) by 2045 (2). Excluding the risk of death associated with the COVID-19 pandemic, an estimated 6.7 million adults died from diabetes or its complications in 2021, which means that approximately one life was lost every 5 seconds (2).

Moreover, diabetic complications, including diabetic foot, are one of the important factors affecting the quality of life of patients with diabetes. Persistent exposure to hyperglycemia means that diabetic foot ulcer (DFU) is mainly caused by peripheral blood vessel disruption and neurological disorders of the lower limb of different degrees in patients with diabetes, which eventually leads to lesions and ulceration in the feet (3). Diabetic foot is the leading cause of hospitalization and is characterized by long hospitalization time, difficult treatment, and expensive medical expenses (4, 5). The risk of developing foot ulcers in patients with diabetes is up to 25% (6) and it has been estimated that a diabetic amputation takes place every 30 seconds around the world (7). Moreover, the cost of treating patients with DFU is four times that of treating patients with non-DFU diabetes (8).

The extremely complex pathological process of the diabetic foot means that routine therapy, such as blood glucose control, surgical vascular bypass, interventional operation, and amputation have certain limitations (9, 10). Scientists have been working to find better treatments. Surprisingly, studies have found that when skin tissue is damaged, new adipocytes will be stimulated to develop and differentiate (11), which indicates the potential of adipose tissue in skin repair. In recent years, adipose tissue (AT) has been revealed as an important endocrine and paracrine organ, which can secrete a wide range of adipokines and extracellular vesicles (EVs) (12–14). And adipose tissue-derived stem cells(ADSCs) are easy to be separated from AT, which are multipotent, self-renewing cells with multidirectional differentiation potential (15–17). Particularly, EVs derived from adipose tissue-derived stem cells(ADSCs-EVs) can uniquely mediate specific target cells through their bio-active cargos such as microRNAs (18). The research about ADSCs-EVs has been surging dramatically in the past 5years, mainly in tissue repair field (19, 20).

This review aims to shed new light on the therapeutic potential of ADSCs-EVs for curing diabetes-induced lower limb ulceration. The identification of the underlying mechanisms by which ADSCs-EVs modulate impaired diabetic wound healing might provide a new strategy for cell-free therapy of diabetic foot ulcers.

## The mechanisms of diabetic non-healing wound development

**Figure 1:**
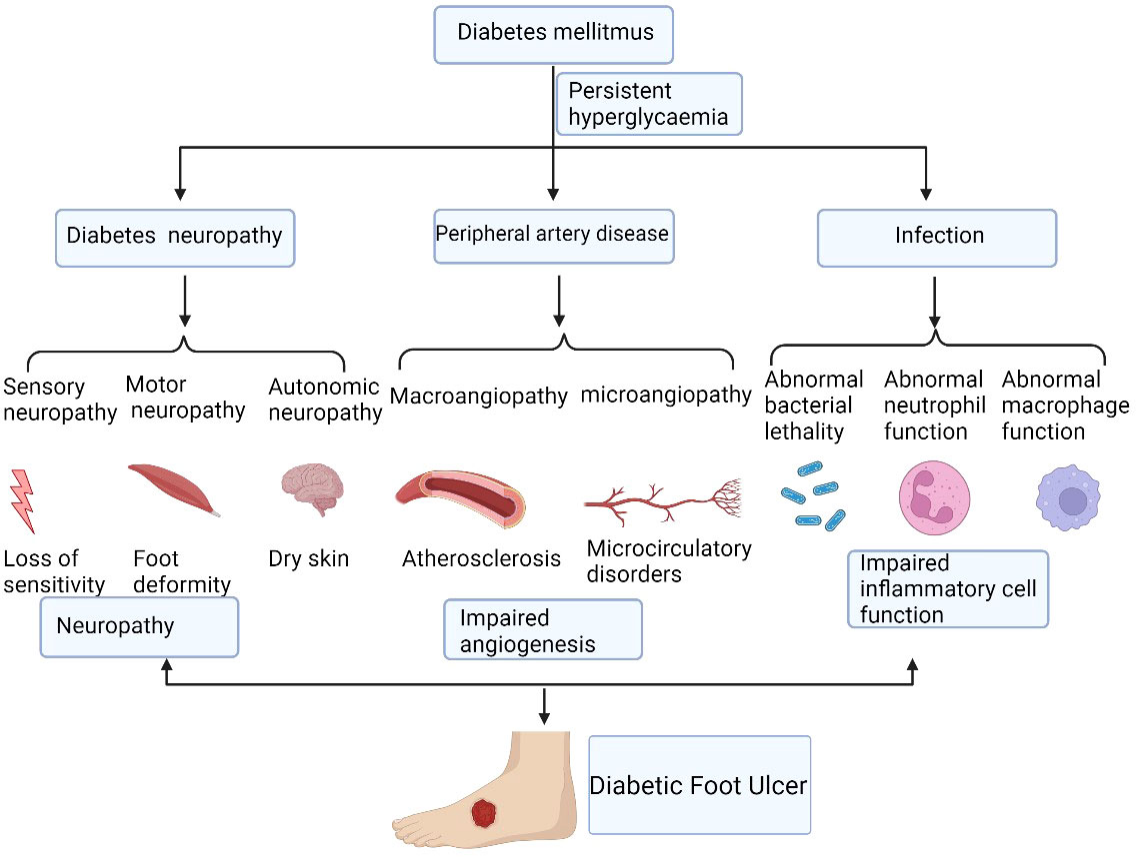
The mechanisms of diabetic non-healing wound development. Diabetic foot ulcers are caused by a number of factors that ultimately lead to chronic wound. These factors include persistent hyperglycaemia, diabetic neuropathy, peripheral artery disease, and local infection, which cause the impairment of angiogenesis and inflammatory cell function. Figure created using BioRender (https://biorender.com/).

Traditionally, acute wound repair is triggered immediately once tissue integrity is disrupted. It is a complex and coordinated process that proceeds through four partly overlapping phases, including hemostasis, inflammation, cellular proliferation, and tissue remodeling, eventually closing the wound (21). Different cells and factors are involved in different stages of wound healing. However, that ability for wound healing in patients with diabetes is impaired and is affected by many factors (**Figure 1**).

Chronic diabetic wounds are initially acute wounds; however, the repair process is interrupted and stagnates at different stages, resulting in delayed healing or chronicity. Currently, the widely accepted viewpoint on the mechanism of DFU is the three-factor theory: diabetic neuropathy, peripheral artery disease, and local infection, in which external minor trauma can act as the inducer to promote the formation and development of ulcers (22).

Diabetic neuropathy presents as a variety of manifestations, affecting sensory, motor, and autonomic nerves (23). Autonomic neuropathy affects 16.7% to 34.3% of patients with diabetes (24). Sometimes it is combined with lesions and becomes a serious complication threatening the lower extremities. It can result in secondary ulcers, infections, and gangrene, requiring amputation or leading to Charcot arthropathy (25). Sensory neuropathy can cause sensory disturbances or painful neuropathy (26). The loss of neurotrophic function can cause muscle atrophy leading to claw toe, crus, foot prolapse, gait change, and gastrocnemius atrophy. Autonomic neuropathy causes changes such as no sweat, dry skin, no hair, and arteriovenous short-circuit and opening (27). Neuropathy can also cause changes to the shape of the feet, known as foot deformities (28). Amputation can also lead to secondary foot deformities, and foot deformities are prone to secondary pressure injuries.

The impaired angiogenesis of diabetes mellitus are caused by both macroangiopathy and microangiopathy (29). For peripheral vascular disease in patients with diabetes, many patients do not have obvious symptoms of diabetes, and severe ischemia of the lower limbs is often the first manifestation of diabetes. Peripheral vascular disease increases the incidence of end-point events in patients with diabetes much more than in patients without diabetes (30, 31).

People with diabetes are more likely to develop any type of infection than people without diabetes (32). Diabetic foot infections are usually caused by trauma and are a major cause of lower extremity risk. They are associated with ulcers and can often lead to amputation. The immunological causes of diabetic podiatry infection comprise abnormal host reactions, including abnormal neutrophil function, abnormal macrophage function, and abnormal bacterial lethality (33, 34). All these factors involves together ultimately causing the impairment of inflammatory cells function (35).

Calluses, blisters, cuts, burns, and inlaid toenails can all lead to DFUs (36). Patients may not be aware of these minor lesions because of peripheral neuropathy; therefore, ulcers might develop and expand before they are detected.

## Overview of extracellular vesicles

**Figure 2:**
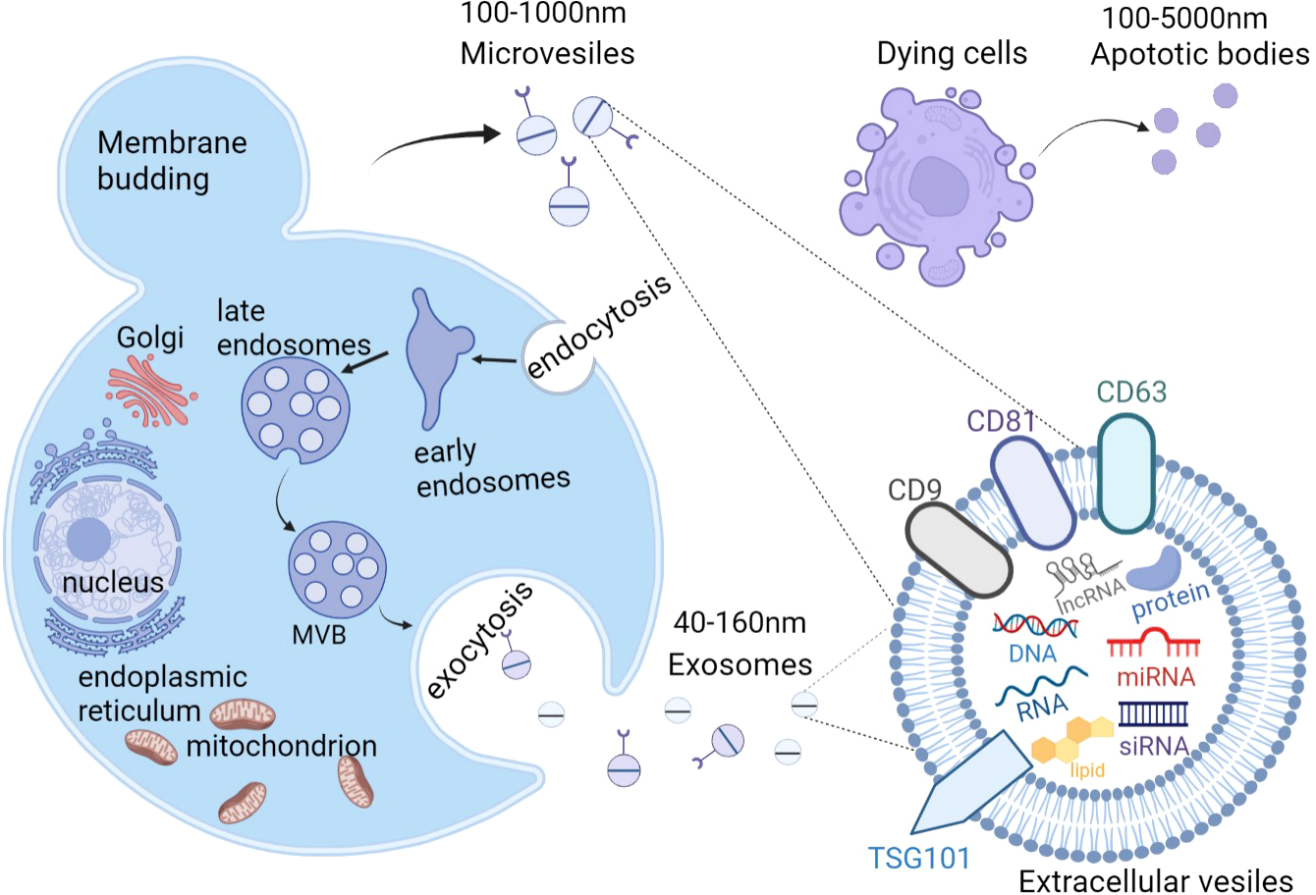
The biogenesis and content of extracellular vesicles(EVs). There are 3 subtypes of EVs, including exosomes, microvesicles (MVs), and apoptotic bodies. Exosomes are generated from the fusion of multivesicular bodies (MVBs) with the plasma membrane, ranging from 40-160nm while MVs are directly produced from the outward budding of the plasma membrane with a diameter of 100-1000nm. Apoptotic bodies are released from the blebbing of dying cells and the diameter is about 100 to 5000 nm. EVs contain proteins, lipids, nucleic acids (DNA, mRNA, siRNA, microRNA, and long noncoding RNAs), and multi-molecular complexes. EVs, extracellular vesicles; MVs, microvesicles; MVBs, multivesicular bodies. Figure created using BioRender (https://biorender.com/).

According to The International Society for Extracellular Vesicles (ISEV), “extracellular vesicle” (EV) has been defined as the generic term as “particles naturally released from the cell that are delimited by a lipid bilayer and cannot replicate, i.e.they do not contain a functional nucleus” (37). Generally, almost all cell types are capable of secreting EVs, and they can be detected in a variety of body fluids, including blood, saliva, semen, lymph fluid, breast milk, urine, amniotic fluid, and cerebrospinal fluid (38). EVs from serum have been investigated as a promising disease biomarker (39).Recently, EVs were identified as intercellular communicators, delivering bioactive cargos, such as proteins, lipids, nucleic acids (DNA, mRNA, microRNAs (miRNAs), and long noncoding RNAs (lncRNAs)) and multi-molecular complexes, further mediating cell-to-cell communication and regulating metabolism and homeostasis (40–44). As heterogeneous cell-derived vesicles, EVs can be roughly divided into exosomes, microvesicles (MVs), and apoptotic bodies according to differences in their size and biogenesis (45) (**Figure 2**). Unlike the production pathway of MVs and apoptotic bodies, exosomes uniquely are created from multivesicular endosomes (MVEs), undergoing inward budding of endosomes and exocytosis, and eventually forming particles with diameter from 40 nm to 160 nm (46). Exosomes(EXOs) are defined by enrichment of specific proteins located on the surface including tetraspanins (CD81, CD63, and CD9) and tumor susceptibility gene 101(TSG101) (47). In contrast, MVs are large vesicles of about 100–1000 nm in diameter, secreting from outward budding of the cell plasma membrane (48–50). As for apoptotic bodies, ranging from 100 to 5000 nm in diameter, they are produced from dying cells through apoptosis or programmed cell death and are released into extracellular space from the plasma membrane (51–53). Recent technological advances have resulted in the emergence of a variety of EVs isolation methods, including ultracentrifugation, sucrose-gradient centrifugation, immunoaffinity bead capture, and size-exclusion chromatography (54). These isolation approaches can be roughly divided into three general categories: density, affinity, and size, according to the principle of their separation mechanisms (55).

However, the recent guideline recommends to using operational terms for EV subtypes that refer to some physical characteristics of EVs, such as size (“small EVs” (sEVs) and “medium/large EVs” (m/lEVs), or biochemical composition such as CD63+/CD81+EVs; or descriptions of conditions or cell of origin like apoptotic bodies. To avoid confusion, we use the term EVs to replace terms such as exosome and microvesicle in this review.

EVs are vesicle structures with double lipid membranes, which have good stability and can protect internal biomolecules from various enzymes in body fluids, thereby maintaining their integrity and biological activity. However, the integrity and biological activity of the extracted exosomes may also be affected by factors such as storage medium, storage temperature and time. At present, the most common storage method is cryopreservation, but cryopreservation may lead to changes in the shape and physical properties of exosomes, and may also lead to the formation and aggregation of multilamellar vesicles, changes in biological properties, content and marker composition. Storage at –80°C is recognized as the most suitable storage environment. Although storage at 4°C can easily lead to the loss of proteins and nucleic acids in exosomes, it can avoid the destruction of vesicles caused by the freeze-thaw process. Compared with 4°C, -70°C and fresh samples, exosomal protein and RNA concentrations were significantly reduced in room temperature storage. Studies have shown that -20°C or lower temperature is a preferable long-term storage condition for exosomes (56). Several reports have shown that acidic pH can reduce the degradation of exosome-related proteins, and that more exosomes can be isolated after conditioned medium or urine with pH adjusted to below 7 for 30 min at room temperature (57).

## Introduction to adipose tissue-derived stem cells-derived extracellular vesicles

Recently, adipose tissue was identified as a primary source of circulating exosomal microRNAs. Adipose-tissue-specific knockout of the miRNA-processing enzyme Dicer (AdicerKO) caused the level of circulating exosomal miRNAs to decrease four-fold (58, 59). As is known to us all, adipocyte hypertrophy is associated with an increased risk for the development of type 2 diabetes (60). It has also been considered to increase the production and release of EVs, which are characterized as the expression of perilipin A (61, 62). Adipose tissue contains abundant stem cells, called adipose tissue-derived stem cells (ADSCs), which are reported to have great potential in wound repair and tissue regeneration. ADSCs-EVs play an important role in this process. ADSCs can secrete a much higher amount of EVs compared to other cell types. EVs derived from different cell types vary in their size and contents. Generally, ADSCs-EVs are mainly distributed into large extracellular vesicles (lEVs) and small extracellular vesicles (sEVs), differing in lipid composition. LEVs are high in phosphatidylserine, while sEVs are high in cholesterol (63).Because EVs have properties similar to parental cells, so ADSCs-EVs contain high content of lipids and lipid droplet-binding proteins such as ATGL and PLIN1 (64).As for function, ADSCs-EVs have been described, primarily as regulators of inflammation and systemic insulin resistance previously (65–68). Recently, a proteomic analysis of extracellular vesicles derived from pig ADSCs revealed the proteins enriched in ADSCs-EVs mainly participated in extracellular matrix remodeling, blood coagulation, inflammation, and angiogenesis (69). Another study found that BMSC-EVs mainly promote cell proliferation and viability, while ADSCs-EVs demonstrated a major effect on endothelial cell migration and angiogenesis (70).

Acting as carriers, ADSCs-EVs, transfer many messages, such as miRNAs, circular RNAs (circRNAs), lncRNAs, and other materials to promote wound repair. Next, we will mainly focus on the mechanism by which ADSCs-EVs promote wound closure.

## The effects of ADSCs-EVs on DFU in experimental models

Several biochemical pathways coordinate skin integrity restoration, in which inflammation is an essential step. Increasing evidence shows that exosomes derived from human ADSCs had significant anti-inflammatory functions *in vitro* wound healing models, thus accelerating wound closure (71). ADSCs secreted exosomes to induce the polarization of macrophages to the M2 phenotype by exosome-carried activated STAT3, thus reducing the ability of macrophages to stimulate the inflammatory response (72). Moreover, ADSCs- EVs expressing a high level of nuclear factor erythroid 2-related factor 2 (Nrf2) decreased the levels of inflammatory cytokines such as IL-1, IL-6, and TNF-α, thus reducing the inflammatory response in the wounds (73).

The proliferative phase serves as the crucial stage of wound healing and mainly involves the proliferation of blood vessels, fibroblasts, and keratinocytes (74). More and more studies have uncovered that ADSCs- EVs are not only able to inhibit cell apoptosis, but also can enhance cell proliferation and angiogenesis. ADSCs - EVs overexpressing nuclear factor erythroid 2-related factor 2 (Nrf2) could advance wound healing by preventing cell senescence and improving vascularization (73). ADSCs- EVs treatment not only significantly suppressed cell apoptosis but also promoted HaCaT cell (a human keratinocyte cell line) proliferation and migration through the Wnt/β-catenin pathway (75). Additionally, the activation of the Wnt/β-catenin signaling pathway plays a profound part in the proliferative phase, the most important event of wound healing (75). Except for that, exosomes from ADSCs can also activate the phosphatidylinositol-4,5-bisphosphate 3-kinase (PI3K)/protein kinase B (AKT) signaling pathway, thus promoting fibroblasts cell proliferation and collagen production (76). Moreover, ADSCs- EVs also serve multiple essential roles in promoting vascular endothelial cells proliferation and migration to accelerate angiogenesis (77). And ADSCs- EVs might also significantly increase skin flaps recovery and capillary density partially through releasing IL-6, therefore repairing ischemia-reperfusion injury (78). Furthermore, ADSCs- EVs can also promote wound healing in some pathological conditions such as hypoxia and high glucose except in normal physiological states. In a mouse model of fat grafting, exosomes derived from human ADSCs under hypoxia conditions enhanced neovascularization partially through the vascular endothelial growth factor (VEGF)/VEGF receptor pathway (79). In addition, human ADSCs-EVs could enhance the cell proliferation, migration, and tube formation of the advanced glycation end product (AGE)-treated human umbilical vein endothelial cells (HUVECs) by activating the PI3K-AKT-mechanistic target of rapamycin (mTOR)-hypoxia-inducing factor alpha (HIF-1α) signaling pathway to motivate repair and angiogenesis of diabetic wound healing (80).

**Figure 3:**
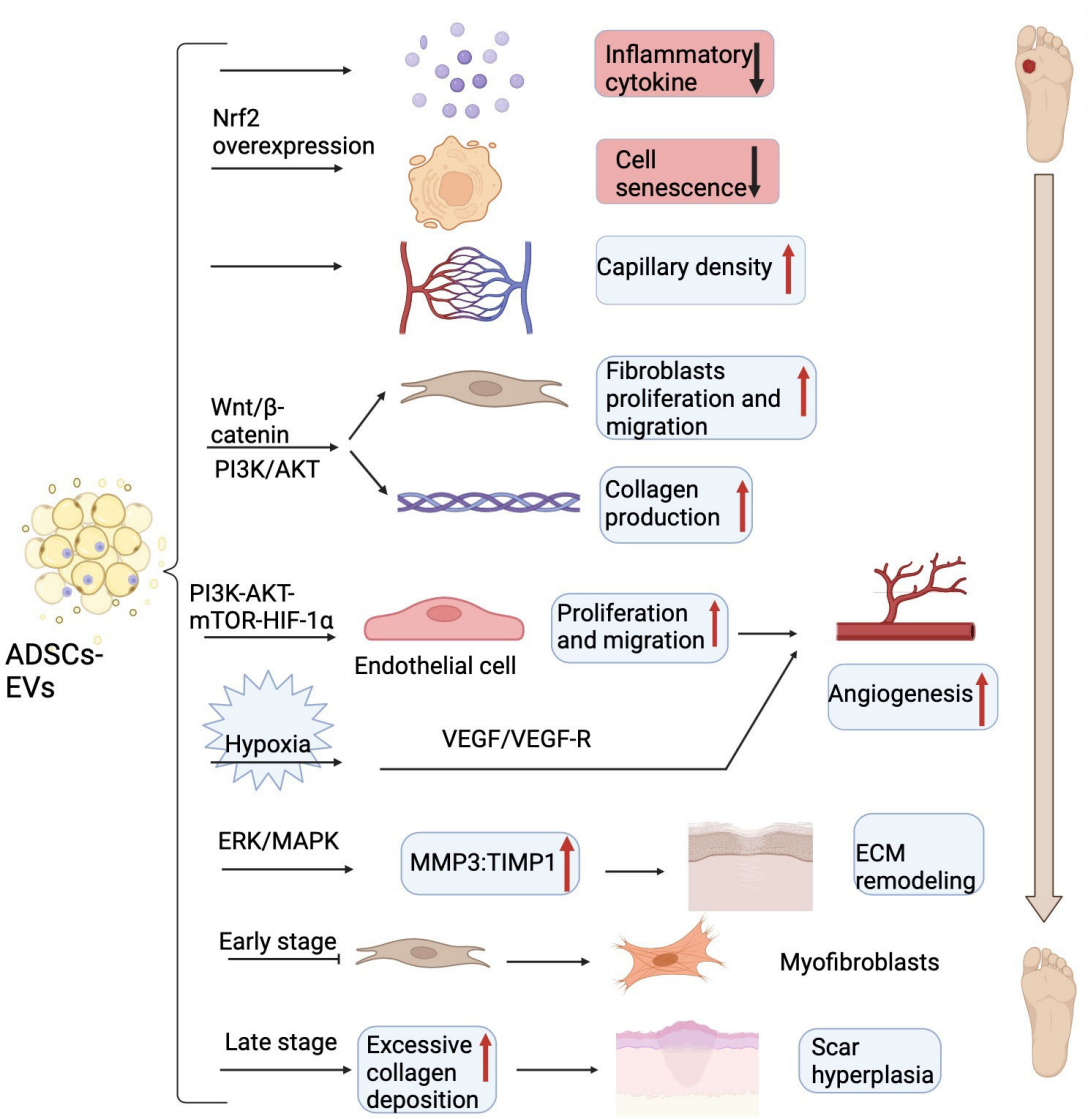
The main mechanism of ADSCs-EVs on DFU in experimental models. ADSCs-EVs can reduce inflammatory cytokines, prevent cell senescence, increase capillary density, promote fibroblasts proliferation and collagen secretion *via* Wnt/β-catenin and PI3K/AKT signaling pathway to accelerate wound closure. ADSCs-EVs also can enhance the endothelial cells proliferation, migration, and tube formation through the PI3K-AKT-mTOR-HIF-1α axis to motivate angiogenesis. Under hypoxia conditions, ADSCs-EVs enhanced neovascularization partially through VEGF/VEGF-R pathway. ADSCs-EVs elevate the ratio of MMP3 to TIMP1 to remodel the extracellular matrix (ECM) and prevent fibroblasts differentiate into myofibroblasts in the early stage and cause excess collagen deposition in the late stage. ADSCs-EVs, adipose tissue-derived stem cells-derived extracellular vesicles; DFU, diabetic foot ulcer; PI3K, phosphatidylinositol-4,5-bisphosphate 3-kinase; AKT, protein kinase B; mTOR, mechanistic target of rapamycin; HIF-1α, hypoxia-inducing factor alpha; VEGF, vascular endothelial growth factor; VEGF-R, vascular endothelial growth factor receptor; MMP3, matrix metalloproteinase 3; TIMP1, tissue inhibitor of matrix metalloproteinases-1; ECM, extracellular matrix. Figure created using BioRender (https://biorender.com/).

Scarring formation is part of the tissue remodeling period of diabetic foot skin damage and the final stage of overall wound healing. Recent research has demonstrated that ADSCs-EVs promote the formation of scarless wounds by preventing fibroblasts differentiate into myofibroblasts. Additionally, ADSCs- EVs also elevated the ratio of matrix metalloproteinase 3 (MMP3) to tissue inhibitor of matrix metalloproteinases-1 (TIMP1) *via* the extracellular regulated kinase (ERK)/mitogen activated protein kinase (MAPK) pathway, thereby remodeling the extracellular matrix (ECM) but also mitigating scar formation (81). However, in a diabetic murine incisional wound model, ADSCs-EVs caused excessive collagen deposition during the wound-healing phase at later stages (82), which would lead to the formation of hypertrophic scar and is detrimental to wound healing (83). Here, we describe the mechanism of ADSCs-EVs regulating wound healing (**Figure 3**).

### ADSCs-EVs -MicroRNAs mediate wound healing

More importantly, microRNAs are enriched in ADSCs-EVs and exosomal microRNAs could reinforce the acceleration of wound healing. ADSCs-EVs contains abundant microRNAs, including microRNA-19b, miR-21, miRNA-31, miRNA-125a, miR-210, miR-486-5p, miR-423-5p, and miR-126-3p (**Table 1**).

**Table 1:** The functions of ADSCs-derived EVs in diabetic foot ulcers.

Non-coding RNAs	Target	Functions	Reference
miR-19b	CCL1	Activate TGF-β pathway, inhibit inflammation, and reduce the apoptosis of cells	(84)
miR-21	TGF-β1	Elevate HaCaT cells migration and proliferation by enhancing the MMP-9 and depressing TIMP-2 expression *via* PI3K/AKT pathway	(85)
miR-31	HIF-1	Promote cell migration and tube formation of HUVECs	(86)
miR-125a	DLL4	Transfer to vascular endothelial cells and promote endothelial tip cell specification to stimulate angiogenesis	(87)
miR-125a-3p	PTEN	Promote the viability, migration, and angiogenesis of HUVECs	(88)
miR-210	RUNX3	Promote HUVECs cell proliferation, migration, and invasion	(89)
miR-486-5p	Sp5	Facilitate fibroblasts proliferation, migration, and HMECs angiogenesis	(90)
miR-423-5p	Sufu	Promote angiogenesis	(91)
miR-126-3p	PIK3R2	Promote proliferation and migration of fibroblasts and angiogenesis of HUVECs	(92)
mmu_circ_0000250	miR-128-3p	Promote SIRT1 expression and enhance angiogenesis	(93)
circ-Gcap14	miR-18a-5p	Upregulate HIF-1α and VEGF expression elevation and angiogenesis	(94)
LncRNA H19	miR-19b	Upregulate SOX9 to activate the Wnt/β-catenin signaling pathway and promote human skin fibroblast cell proliferation, migration and invasion	(95)
Linc00511	PAQR3	Upregulate Twist1 and EPCs proliferation, migration, and angiogenesis	(96)
LncRNA MALAT1	miR-124	Activate Wnt/β-catenin pathway, thereby promoting cutaneous wound healing	(97)

miR/miRNA, microRNA; CCL1, C-C motif chemokine ligand 1; TGF-β, transforming growth factor beta; TGF-β1, transforming growth factor beta1; MMP-9, matrix metalloproteinase 9; TIMP-2, tissue inhibitor of matrix metalloproteinases-2; PI3K, phosphatidylinositol-4,5-bisphosphate 3-kinase; AKT, protein kinase B; HIF-1, hypoxia-inducing factor alpha; DLL4, delta-like 4; PTEN, phosphatase and tensin homolog; RUNX3, RUNX family transcription factor 3; Sp5, Sp5 transcription factor; Sufu, suppressor of fused homolog; PIK3R2, phosphoinositide-3-kinase regulatory subunit 2; circ-, circRNA; SIRT1, sirtuin 1; VEGF, vascular endothelial growth factor; SOX9, SRY-box transcription factor 9; PAQR3, progestin and adipoQ receptor family member 3; MALAT1, metastasis-associated lung adenocarcinoma transcript 1.

miR-19b derived from ADSCs-EVs mediates the transforming growth factor beta (TGF-β) pathway by targeting CCL1 (encoding C-C motif chemokine ligand 1) (84). In addition, incubation with ADSCs or their derived exosomes could prevent the increased HaCaT cell apoptosis rate,and meanwhile, an *in vivo* mouse model of skin damage further confirmed that miR-19b could significantly promote the process of the healing of cutaneous damage (84). High levels of miR-21 were found in exosomes derived from ADSCs and could elevate the HaCaT cells cell migration and proliferation by enhancing MMP-9 and depressing TIMP-2 levels *via* the PI3K/AKT pathway (85). At the same time, this study also showed that overexpression of miR-21 could suppress TGF-β1 expression and excess TGF-β1 had a negative feedback influence on miR-21 (85). For instance, a study found that MVs were able to increase the migration and tube formation of HUVECs. Moreover, miR-31 could promote cell migration and tube formation of HUVECs by targeting the antiangiogenic gene HIF1, thus contributing to the proangiogenic effect (86). miR-125a is also enriched in exosomes secreted from human ADSCs and can be absorbed into vascular endothelial cells through exosomes, thus enhancing angiogenesis (87). Additionally, miR-125a directly represses its downstream target gene *DLL4*, encoding delta-like 4, an angiogenic inhibitor, thus promoting endothelial tip cell specification and modulating endothelial cell angiogenesis (87). Another study revealed that miR-125a-3p from human ADSCs -EVs promoted wound healing and angiogenesis by inhibiting *PTEN* (encoding phosphatase and tensin homolog (88). In addition, miR-210 released from ADSCs -derived MVs, which was overexpressed under hypoxia, promoted HUVEC proliferation and migration by directly targeting *RUNX3* (encoding RUNX family transcription factor 3) *in vivo* and *in vitro (* 89). Another study showed that miR-486-5p secreted from ADSCs-EVs promotes angiogenesis and expedites the healing progression of cutaneous wounds by inhibiting the expression of *SP5* (encoding Sp5 transcription factor) (90). And exosomal miR-423-5p derived from human- ADSCs can be transferred into HUVECs and promote angiogenesis by targeting (suppressor of fused homolog)Sufu (91). Recent studies have demonstrated that there are 199 upregulated miRNAs and 93 downregulated miRNAs in ADSCs-EVs compared to the ADSCs group, stimulating dermal fibroblast proliferation and migration to promote skin regeneration (98). For instance, ADSCs-derived exosomal miR-126-3p not only promoted fibroblasts proliferation and migration but also stimulated angiogenesis by targeting *Pik3r2* (encoding phosphoinositide-3-kinase regulatory subunit 2), thus accelerating collagen deposition, new vessel formation, and wound healing in the rats model (92). Another research also found that reduced miR-126 in serum-EVs impaired the angiogenic potential of endothelial cells (99).

### ADSCs-EVs -circRNAs mediate wound healing

Recent studies have provided strong evidence that circRNAs play a key role in regulating wound repair microenvironments (100) (**Table 1**). A recent study reported that in a high glucose pathological environment, angiogenesis is inhibited, while mmu_circ_0000250 could enhance angiogenesis in a model of angiogenesis *in vitro (* 93). Overexpression of mmu_circ_0000250 suppressed miR-128-3p expression, thereby increasing the expression level of *SIRT1* (encoding sirtuin 1), which has anti-inflammatory and antioxidant features (101, 102). Moreover, in a DFU mouse model, consistent with previous results, exosomes containing abundant mmu_circ_0000250 could largely accelerate the process of wound healing (93). Another study identified that circ-Gcap14 was upregulated in hypoxic preconditioned ADSCs (94). The study confirmed that circ-Gcap14 could act as a microRNA sponge to absorb miR-18a-5p, resulting in the upregulation of HIF-1α and subsequent VEGF expression elevation, thereby promoting angiopoiesis and wound healing (94).

### ADSCs-EVs - lncRNAs mediate wound healing

Many studies reported ADSCs-EVs to comprise varieties of lncRNAs, including *H19*, Linc00511, and lncRNA MALAT1 (**Table 1**). LncRNA *H19* functions as a molecular sponge for miR-19b directly targeting *SOX9* (encoding SRY-box transcription factor 9), so ADSCs-EVs with an overabundance of *H19* suppressed miR-19b levels leading to *SOX9* upregulation, which activated the Wnt/β-catenin signaling pathway. And this activation could promote the cell proliferation, migration, and invasion of human skin fibroblast and accelerate wound healing of skin tissues (95). *Linc00511*-overexpressing ADSCs-EVs upregulated Twist1 expression by repressing Twist1-ubiquitination and degradation *via* inhibition of progestin and adipoQ receptor family member 3 (PAQR3) in endothelial progenitor cells (96). ADSCs could also secrete exosomes containing lncRNA MALAT1 (metastasis-associated lung adenocarcinoma transcript 1), which are capable of promoting human dermal fibroblast migration and accelerating wound healing (103). Furthermore, the subsequent study found that ADSCs- EVs containing MALAT1 could target miR-124 and activate Wnt/β-catenin pathway, thereby promoting cutaneous wound healing (97).

### ADSCs-EVs loaded with other molecules mediate wound healing

Studies have shown that MSC-EVs mainly act *via* their encapsulated miRNAs; however, other studies have shown that EVs can act independently of miRNAs. For example, a previous study showed that miR-205 could modulate AKT activation, thereby increasing keratinocyte migration and facilitating cutaneous wound repair (104). However, interestingly, later research reconfirmed the capacity of ADSCs -EVs to accelerate cell proliferation and migration, but surprisingly, this effect of ADSCs -EVs on wound healing was independent of miR-205 activity (105). Furthermore, the study also found that knockdown of miR-205 inhibited AKT phosphorylation in fibroblasts and keratinocytes, and administration of ADSCs-EVs reversed the effect caused by miR-205 knockdown, meanwhile, an *in vivo* animal experiment proved that ADSCs-EVs promoted skin wound closure in a manner independent of miR-205 activity (105). Additionally, many other compositions like proteins also play a great role in curing DFU. Another study showed that ADSCs- EVs contain angiopoietin like 1 (ANGPTL1), thrombopoietin, and milk fat globule EGF and factor V/VIII domain containing (MFGE8), which have angiogenic effects (106). A recent study has found that ADSCs -EVs are particularly rich in pro-angiogenic genes and NRG1(neuregulin 1), and ADSCs -EVs can promote angiogenesis and prevent muscle inflammation cells infiltration *via* NRG1-mediated signals under ischemia/reperfusion condition (107). EVs derived from endothelial cells were reported to inhibit vascular smooth muscle cell apoptosis and increase recruitment to neovessels *via* carried PDGF-BB (108).

## The potential clinical application of ADSCs-EVs

In conclusion, ADSCs -EVs have great potential to cure diabetic wounds, representing a novel potential therapy to treat chronic wounds. In the current view, existing treatments for DFU mainly include glycemic control, nutritional support, drug therapy, pressure offloading, vascular reconstruction, surgical debridement, and stem cell therapy (109). However, these available therapeutic methods and options are very limited and none of the above are adequately powered to cure diabetic foot. Thus, scientists have attempted to develop an ideal therapy by applying ADSCs -EVs to cure DFU, mainly including ADSCs-EVs injection therapy, EVs -loaded alginate hydrogel, and EVs -loaded wound dressings.

### ADSCs- EVs injection therapy

A recent study has applied ADSCs- EVs to wounds through topical injection and intravenous injection and interestingly found that when given intravenous injection treatment, the wound healed faster compared to local injection (83). On the one hand, the different results caused by two injection ways may lie in partial loss of exosomes during the local injection. On the other hand, when exosomes are injected directly into the wound, they unavoidably further impact the wound, thereby disrupting the healing process (83). Another research has illustrated that a combination intravenous administration of human ADSCs and human ADSCs- EVs could significantly enhance cutaneous regeneration, collagen deposition, and angiogenesis in a mouse cutaneous wound healing model for the first time (110). In addition, for local application, human ADSCs- EVs offered additional benefits for wound healing over human ADSCs (110).

### EVs -loaded alginate hydrogel

EVs are commonly administered by injection, which is reported to undergo a fairly rapid systemic clearance thus impacting their function (111). However, the capability of diabetic wound repair and tissue restoration is impaired, which means a relatively prolonged healing time. More recently, hydrogels have been extensively applied in the tissue engineering and regenerative medicine fields due to their diverse characteristics, including supporting the incorporation of therapeutic cells (112). Studies indicated that EVs can be delivered using hydrogels, which enhanced their angiogenic activities and facilitated wound healing (113, 114). Chitosan-based hydrogels loaded with EVs have been heavily exploited to restore vascularization and also promote the wound healing process (115). In addition, multifunctional hydrogels have been developed. One type of multifunctional hydrogel is called FHE hydrogel (F127/OHA-EPL), consisting of Pluronic F127 (F127), oxidative hyaluronic acid (OHA), and ϵ-poly-L-lysine (EPL). Recent studies have found that an FHE@exosomes (FHE@exo) hydrogel significantly promoted the proliferation, migration, and tube formation ability of HUVECs. Meanwhile, the FHE@exo hydrogel significantly accelerated angiogenesis, re-epithelization, and collagen deposition and enhanced diabetic wound healing with less scar tissue (116). Apart from FHE hydrogel, alginate-based hydrogels have received great attention because of their high biocompatibility and capacity for sustained release of their carried bioactive molecules (117–119). The application of ADSCs- EVs incorporated into alginate hydrogels significantly promotes active wound closure, reepithelization, collagen deposition, and angiogenesis in cutaneous full-thickness wounds in a rat model (120). Chitin nanofiber hydrogel obtained from squid cartilage has also received attention because it can largely simulate the natural ECM, promote cell inoculation, and absorb the wound exudate from the wound (121). Another study showed that an ADSCs-loaded β-Chitin nanofiber hydrogel could significantly promote vessel formation and collagen deposition *via* the TGFβ/SMAD family member 3 (smad3) pathway, thus promoting the wound healing process (122).

### EVs -loaded wound dressing

Recently, regenerative wound dressings have become a trending topic in the tissue repair field, because they are biodegradable and will eventually integrate with the wound after serving as a substrate for tissue to form (123). One kind of EVs -loaded oxygen releasing antioxidant wound dressing, OxOBand, contributed to faster re-epithelialization, improved angiogenesis, and enhanced collagen synthesis, ultimately accelerating wound repair and tissue regeneration in an *in vivo* diabetic wound model. OxOBand consists of antioxidant polyurethane, which releases oxygen persistently and can be supplemented with ADSCs-EXOs (123).

Importantly, these dressings have the potential to prevent infection and ulceration, improve wound healing with increased collagen deposition, and promote re-epithelialization, Thus, OxOBand is a remarkable new therapy to enhance diabetic wound healing and might provide a promising therapeutic strategy to treat diabetic ulcers (123).

Human acellular amniotic membrane (hAAM), which is readily available and inexpensive, has been reported to have significant potential as a scaffold dressing to promote would repair (124–126). A recent study proved that a combination of hAAM and ADSCs- EVs enhanced wound repair by mediating inflammation, promoting angiogenesis, and advancing the synthesis of the ECM. The hAAM- EVs scaffold dressing is a novel non-invasive approach to delivering exosomes rather than a kind of wound dressing (127). The FEP scaffold was constructed using F127, grafting polyethylenimine and aldehyde pullulan, which comprises an adhesive thermosensitive multifunctional dressing with a long-term exosome-release property. FEP scaffolds loaded with human ADSCs-exosomes (FEP@exo) synergistically accelerated fast and scarless healing by stimulating angiogenesis and enhancing cell proliferation and re-epithelialization. In addition, FEP@exo exerted better effects than the exosome solution alone, indicating great power in wound healing (128). A recent study has proposed a scaffold named dipose-derived stem cells (ADSCs) loaded gelatin-sericin (GS) coated with laminin (GSL) cryogels, which has an effect on more vascularization and could improve healing in compromised chronic wounds (129). Meanwhile, an asymmetric wettable dressing with a composite of exosomes and silver nanoparticles (CTS-SF/SA/Ag-Exo dressing) was fabricated to solve the repair of infected wounds, possessing multifunctional properties including broad-spectrum antimicrobial activity, promoting wound healing, retaining moisture and maintaining electrolyte balance (130).

## Discussion and conclusion

EVs contain abundant content such as non-coding RNAs, proteins, and lipids. Apart from non-coding RNAs carried by EVs, there are some non-coding RNAs that exist in free form or binding form to the protein particles in circulation. Most of the endogenous circulating miRNA molecules do not exist in free form, but often exist in particles formed with proteins, so the endogenous circulating RNA molecules have good RNase degradation resistance and high stability. But non-coding RNAs in EVs show remarkable stability and more durable activity, and have good RNase degradation resistance. And a higher expression of the EV-derived miRNA pool is likely to result from a shell-like protective activity exerted by the EVs on miRNAs in plasma (131). More importantly, among the EV bioactive cargoes, non-coding RNAs in EVs are an important component of gene regulation, eliciting either decay or translational repression of target mRNAs while free non-coding RNAs do not have it (132). EVs secreted by ADSC contain a variety of proliferation-promoting miRNA, lncRNA, cytokines and active peptide substances, and are wrapped by lipid bilayer membrane, so it is difficult to be decomposed and more easily transferred to target cells to play functions. As is known to us all, there are three different kinds of fat depots, their functions are different. However,there is no literature on the therapeutic effect of brown fat, beige fat, and white adipose-derived EVs on diabetic foot ulcer so far. So our review only focuses on general ADSCs, just want to see the all ADSCs-EVs influence on DFU, but the specific mechanism needs more investigation. ADSCs -EVs can promote tissue regeneration and repair by regulating cell proliferation and apoptosis, and participate in the regulation of angiogenesis, immune regulation, and ECM remodeling. Remarkably, many pre-clinical data support the view that ADSCs -EVs therapy has immunomodulatory and reparative properties which accelerate diabetic wound healing; therefore, they are expected to be a novel and better treatment for DFU. In sum, adipose-derived stem cells (ADSCs) represent an ideal resource for stem cell-based regenerative medicine, which are characterized by accessibility, multipotency, self-renewal potential, immune-privilege, and high proliferative rate. ADSCs possess great power, especially in potentially curing diabetic foot ulcers. Considerable researches indicate that ADSCs -EVs can promote tissue regeneration and repair by regulating cell proliferation and apoptosis, and participating in the regulation of angiogenesis, immune regulation, and extracellular matrix remodeling.

ADSCs-EVs have great potential, but there are also many obstacles. First of all, the preparation of ADSCs-EVs is time-consuming and complicated. Additionally, the extraction quantity of EVs is small and the efficiency is low. Moreover, existing extraction schemes still cannot meet the clinical standards and needs (133). Therefore, a safe and efficient approach needs to be further developed to obtain a great deal of EVs. And lack of suitable storage and transport methods to ensure the stability of EVs is another headwind to deal with. EVs are generally stored at -80°C, but this temperature is not suitable for the processing or transportation of EVs. More importantly, long-term storage of exosomes at -80°C will lead to its morphology alterations, decreased biological activity, and RNA degradation (134). Current studies on adipose stem cell-derived exosomes are mainly short-term studies or small sample studies, lacking long-term clinical studies (135, 136). Nevertheless, the pathway of preclinical experimental models to clinical application is so long and hard, because the challenges will be numerous. Still, if we can figure out the detailed mechanism of ADSCs -EVs in promoting wound healing, the forward road will be easier.

## Author contributions

HD and YC wrote the manuscript. YC edited the manuscript. All authors contributed to the article and approved the submitted version.

## Funding

This study was supported by the National Natural Science Foundation of China (82070859 to YC) and a grant from Tongji Hospital in Huazhong University of Science and Technology (Grant No. 2201103295 to YC).

## Conflict of interest

The authors declare that the research was conducted in the absence of any commercial or financial relationships that could be construed as a potential conflict of interest.

## Publisher’s note

All claims expressed in this article are solely those of the authors and do not necessarily represent those of their affiliated organizations, or those of the publisher, the editors and the reviewers. Any product that may be evaluated in this article, or claim that may be made by its manufacturer, is not guaranteed or endorsed by the publisher.
